# Supplementary material for: Therapeutic Potential of (−)-Agelamide D, a Diterpene Alkaloid from the Marine Sponge Agelas sp., as a Natural Radiosensitizer in Hepatocellular Carcinoma Models
Source: Mar Drugs. 2020 Sep 29;18(10):500. doi: 10.3390/md18100500 (PMC7600430; doi:10.3390/md18100500)
Supplement: Supplementary file 1 [file marinedrugs-18-00500-s001.pdf]

marine drugs

Supplementary Materials

Therapeutic Potential of (−)-Agelamide D, a Diterpene Alkaloid from the Marine Sponge *Agelas* sp., as a Natural Radiosensitizer in Hepatocellular Carcinoma Models

Changhoon Choi [1], Yeonwoo Cho [2,3], Arang Son [1], Sung-Won Shin [1,4], Yeon-Ju Lee [2,3,*], and Hee Chul Park [1,4,*]

[1] Department of Radiation Oncology, Samsung Medical Center, Seoul 06351, Korea; chchoi93@gmail.com (C.C.); onlyshohow@naver.com (A.S.); camuserik@gmail.com (S.-W.S.)

[2] Marine Natural Products Chemistry Laboratory, Korea Institute of Ocean Science and Technology, 385 Haeyangro, Busan 49111, Korea; yeonwoo@kiost.ac.kr

[3] Department of Applied Ocean Science, University of Science and Technology, Daejeon 34113, Korea

[4] Department of Radiation Oncology, Sungkyunkwan University School of Medicine, Seoul 06351, Korea

* Correspondence: yjlee@kiost.ac.kr (Y.-J.L.); hee.ro.park@samsung.com (H.C.P.)

Tables of Contents

Table S1. Serum biochemical parameters in two groups of mice that received radiation therapy (RT) or RT plus (−)-agelamide

Figure S1. Underwater (**A**) and *in situ* (**B**) photographs of *Agelas* sp. used in this study

Figure S2. Bioassay-guided isolation of compounds **1** and **2** from *Agelas* sp.

Figure S3. Enhancement of apoptosis by combined treatment with fractions from *Agelas* extract and radiation

Figure S4. ^{1}H and ^{13}C NMR spectra (600 MHz and 150 MHz, methanol-d_3) of compound **2**

Figure S5. COSY and HSQC spectra of compound **2**

Figure S6. HMBC spectra of compound **2**

Figure S7. Selective COSY and HMBC correlations for compound **2**

Table S1. Serum biochemical parameters in two groups of mice that received radiation therapy (RT) or RT plus (−)-agelamide

Parameters	Unit	RT	RT plus (−)-agelamide
Aspartate transaminase	U/L	81.0 ± 26.3	81.3 ± 7.1
Alanine transaminase	U/L	28.7 ± 2.1	26.0 ± 4.4
Alkaline phosphatase	U/L	99.3 ± 20.2	91.0 ± 11.5
Total protein	g/dL	4.8 ± 0.5	4.9 ± 0.2
Albumin	g/dL	2.6 ± 0.3	2.7 ± 0.2
Total bilirubin	mg/dL	0.1	0.1
Blood urea nitrogen	mg/dL	22.2 ± 4.0	25.6 ± 1.8
Creatine	mg/dL	0.5 ± 0.1	0.5
Glucose	mg/dL	247.7 ± 74.7	224.7 ± 64.1
Cholesterol	mg/dL	69.0 ± 3.6	74.7 ± 7.6
Triglyceride	mg/dL	65.0 ± 4.6	70.3 ± 14.6
Phosphorus	U/L	7.4 ± 1.6	5.6 ± 1.0
Ca^{2+}	mg/dl	8.8 ± 0.7	8.5 ± 0.3
Creatine phosphokinase†	U/L	232.1 ± 69.0	485.4 ± 147.3
A/G ratio		1.2 ± 0.2	1.3 ± 0.3

†Statistically not significant

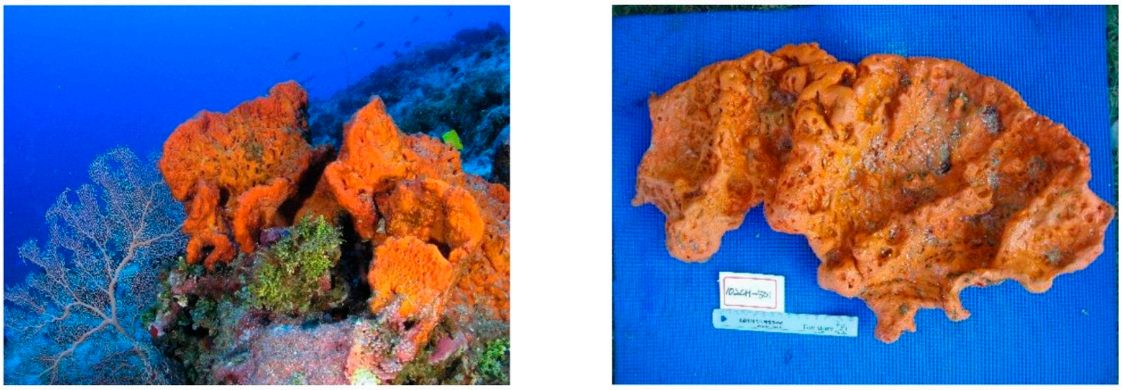

A **B**

Figure S1. Underwater (**A**) and *in situ* (**B**) photographs of *Agelas* sp. used in this study

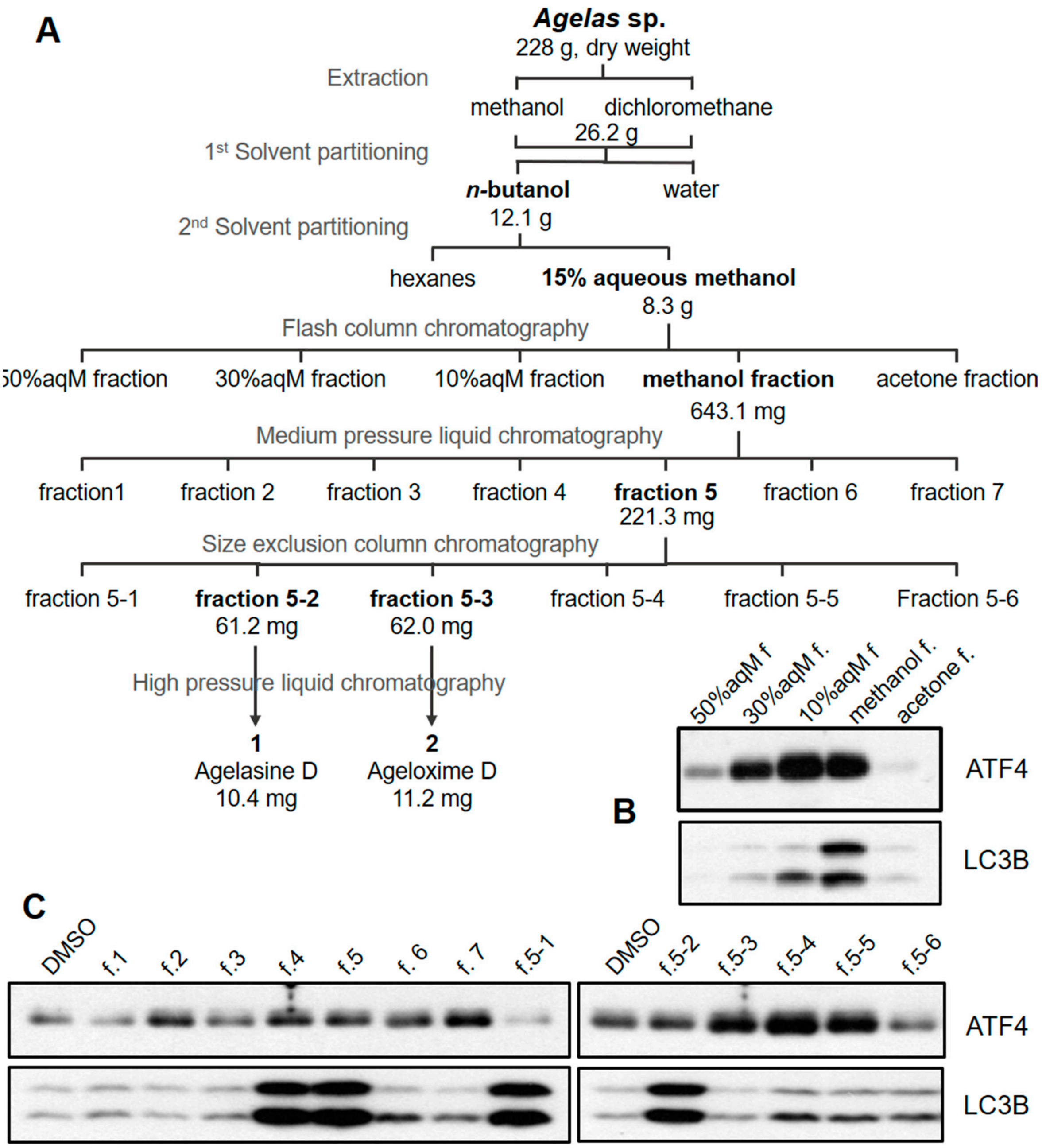

Figure S2. Bioassay-guided isolation of compounds 1 and 2 from *Agelas* sp. **A,** Extraction and isolation scheme for compounds 1 and 2 from *Agelas* sp. **B,** Levels of ATF4 and LC3B induced by the fractions obtained through flash column chromatography. **C,** Levels of ATF4 and LC3B induced by the fractions obtained through medium pressure liquid chromatography (MPLC) (fractions 1–7) and size exclusion column chromatography (fractions 5.1–5.6).

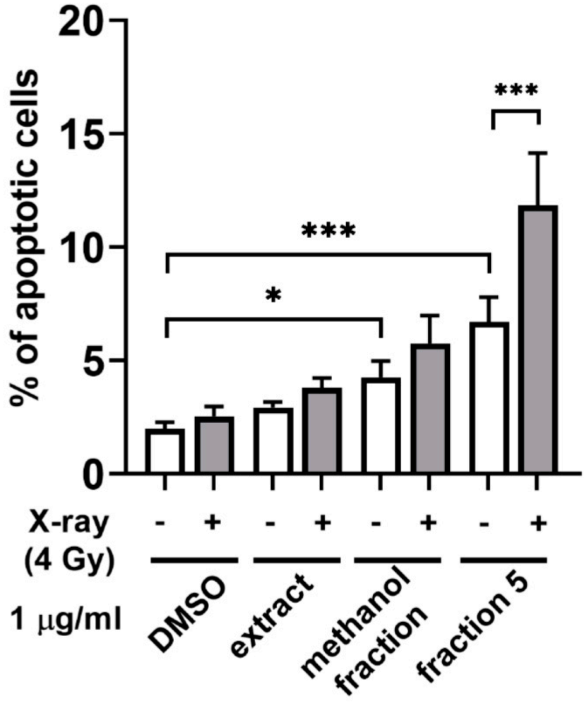

Figure S3. Enhancement of apoptosis by combined treatment with fractions from *Agelas* extract and radiation. Hep3B cells were pre-treated with the same amount (1 μg/mL) of the samples from each purification step for 3 h, followed by irradiation with 4 Gy of X-rays. After 48 h, the samples were collected, and the apoptotic cell population was analyzed through flow cytometry with Annexin V/propidium iodide co-staining. Data are mean ± standard deviation (SD) from two independent experiments ($n = 6$). * $p < 0.05$; *** $p < 0.001$.

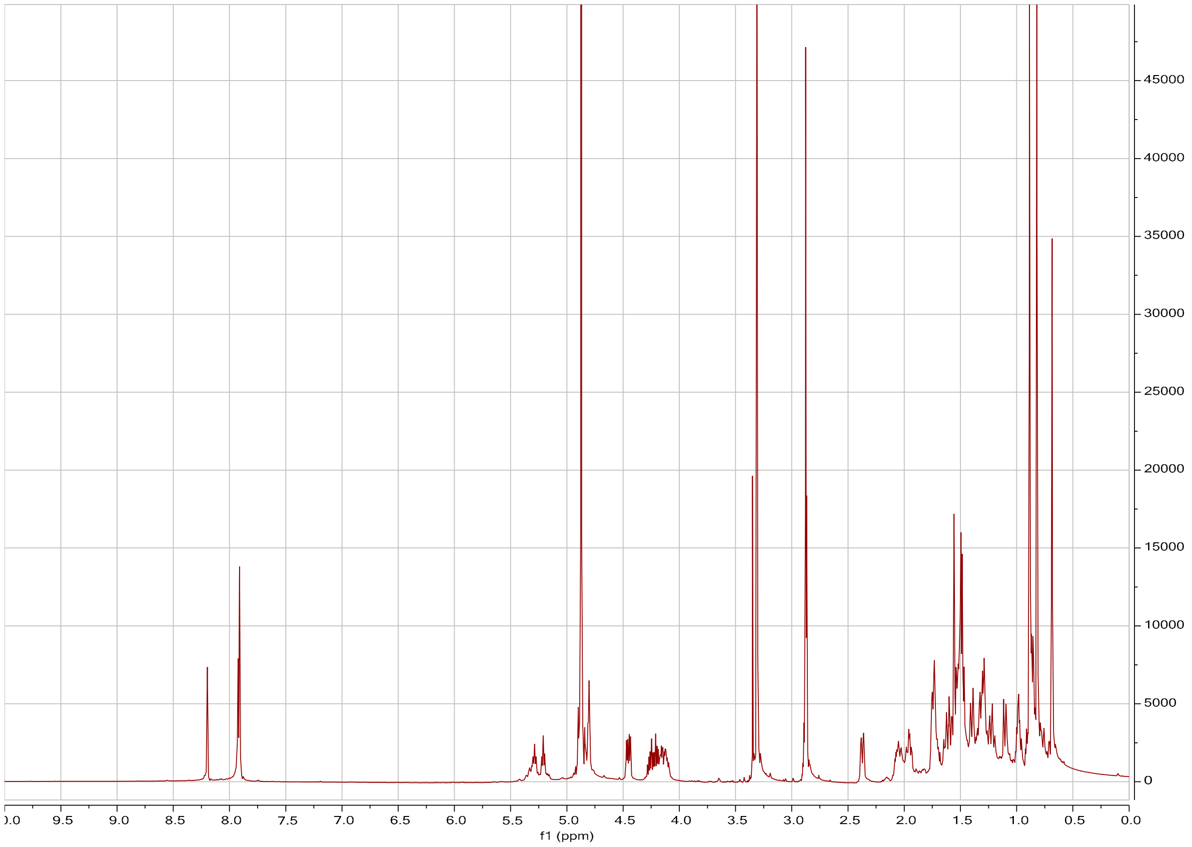

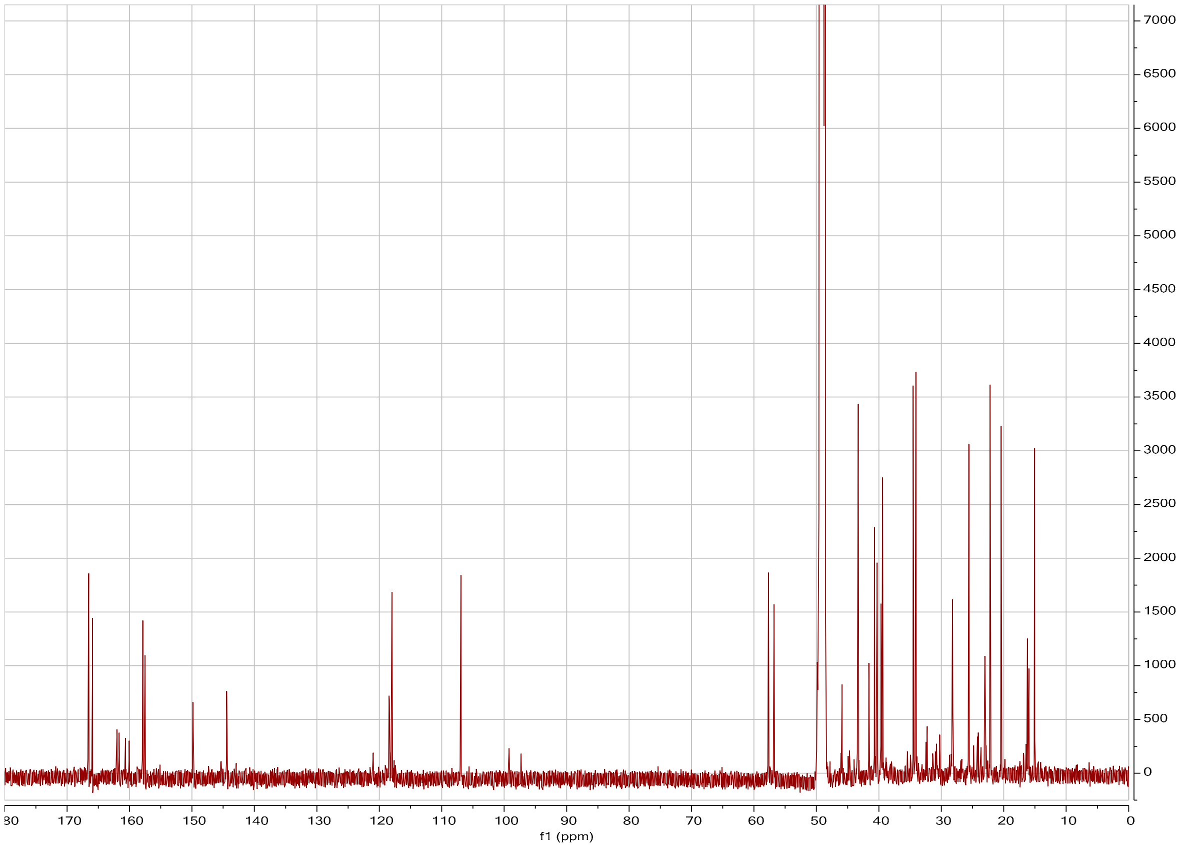

Figure S4. ^{1}H and ^{13}C NMR spectra (600 MHz and 150 MHz, methanol-d_4) of compound **2**

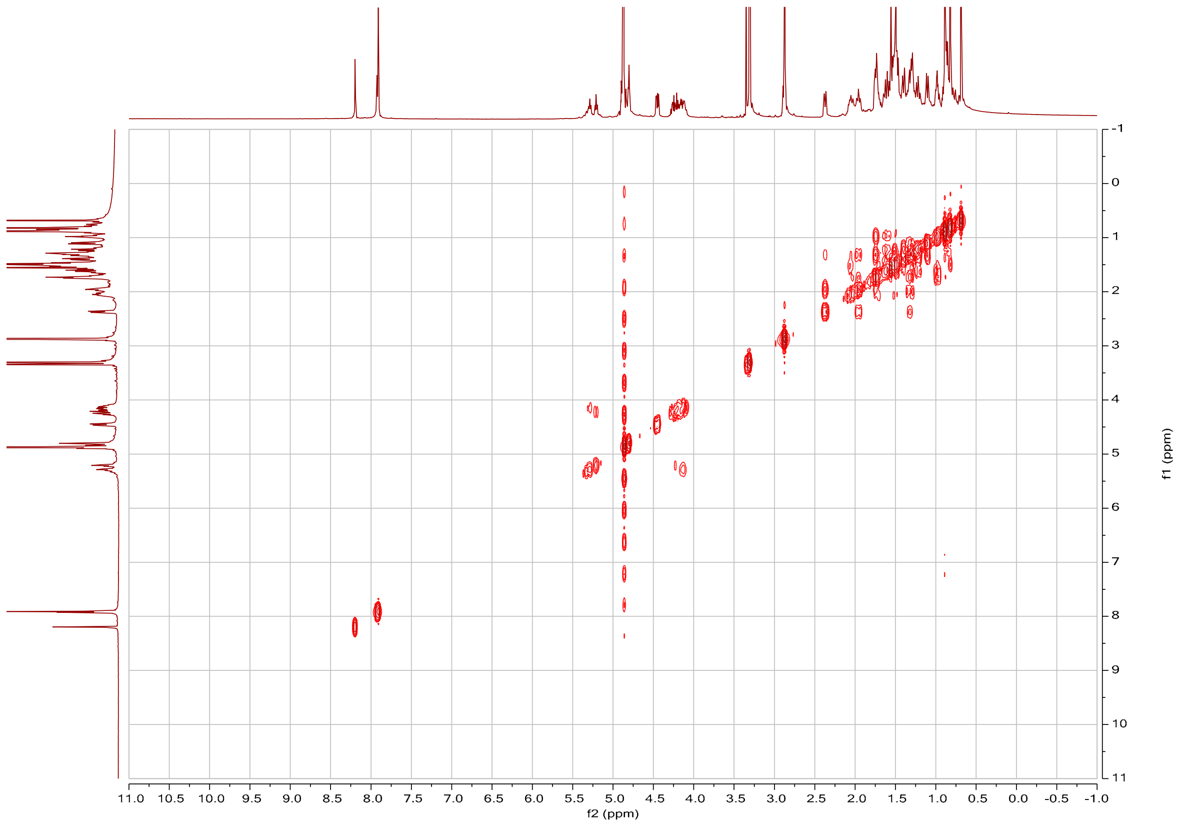

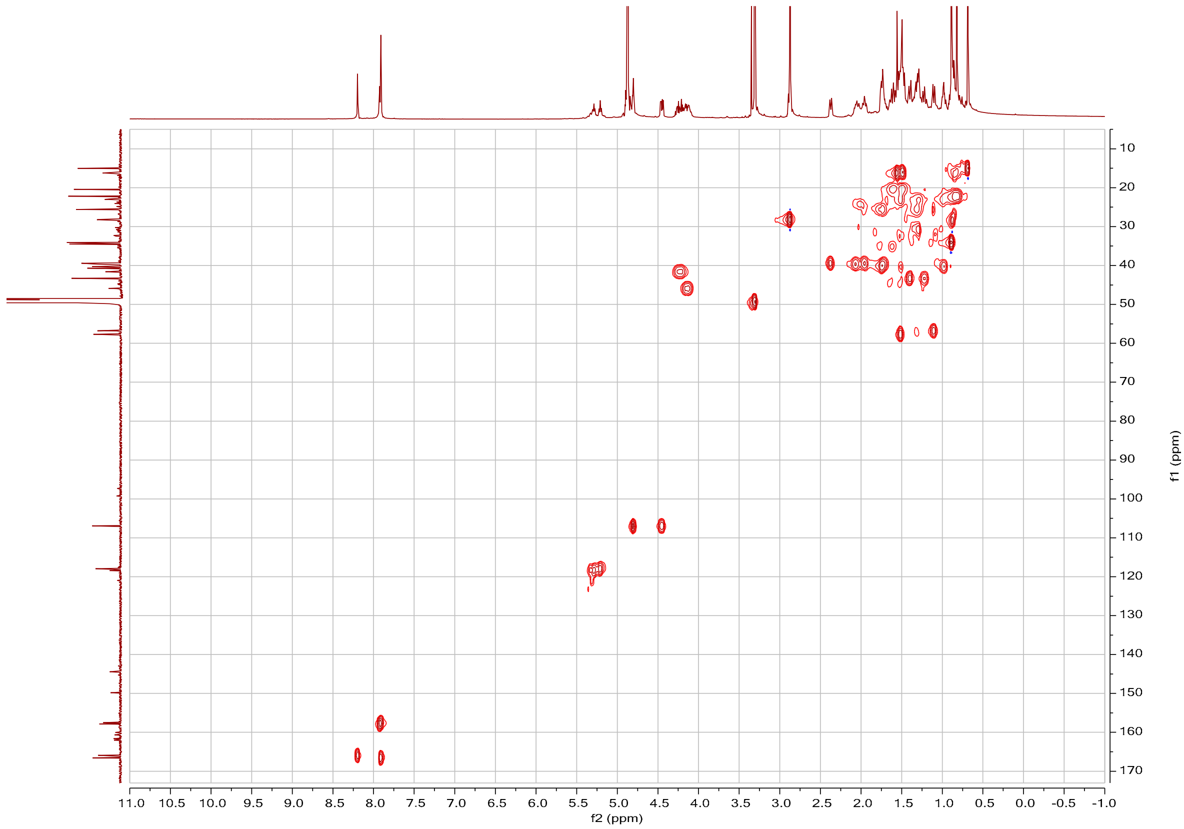

Figure S5. COSY and HSQC spectra of compound **2**

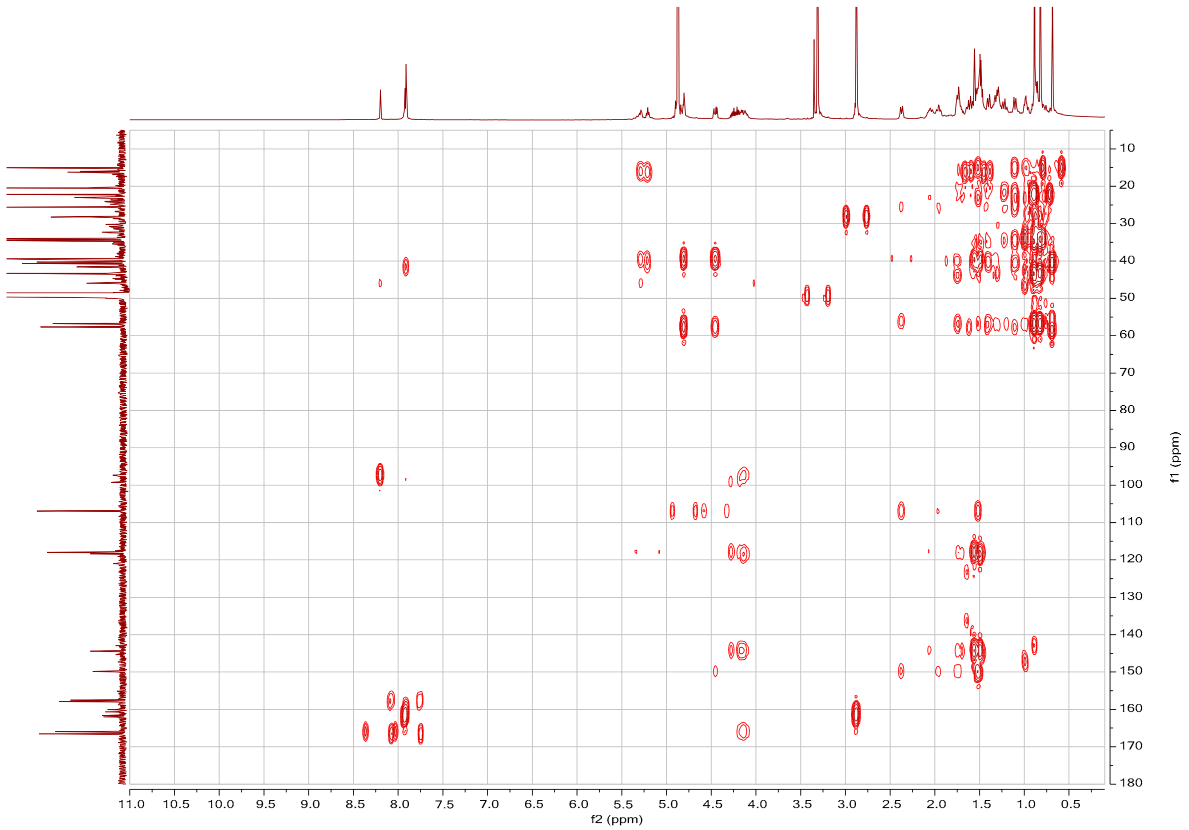

Figure S6. HMBC spectra of compound **2**

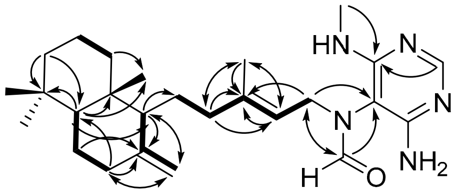

Figure S7. Selective COSY and HMBC correlations for compound **2**
